# Acute resistance exercise modulates microRNA expression profiles: Combined tissue and circulatory targeted analyses

**DOI:** 10.1371/journal.pone.0181594

**Published:** 2017-07-27

**Authors:** Randall F. D’Souza, James F. Markworth, Kirsten M. M. Aasen, Nina Zeng, David Cameron-Smith, Cameron J. Mitchell

**Affiliations:** 1 Liggins Institute, The University of Auckland, Auckland, New Zealand; 2 Department of Orthopedic Surgery, University of Michigan, Ann Arbor, Michigan, United States of America; Deakin University, AUSTRALIA

## Abstract

A subset of short non-coding RNAs, microRNAs (miRs), have been identified in the regulation of skeletal muscle hypertrophy and atrophy. Expressed within cells, miRs are also present in circulation (c-miR) and have a putative role in cross-tissue signalling. The aim of this study was to examine the impact of a single bout of high intensity resistance exercise (RE) on skeletal muscle and circulatory miRs harvested simultaneously. Resistance trained males (n = 9, 24.6 ± 4.9 years) undertook a single bout of high volume RE with venous blood and muscle biopsies collected before, 2 and 4hr post-exercise. Real time polymerase chain reaction (Rt-PCR) analyses was performed on 30 miRs that have previously been shown to be required for skeletal muscle function. Of these, 6 miRs were significantly altered within muscle following exercise; miR-23a, -133a, -146a, -206, -378b and 486. Analysis of these same miRs in circulation demonstrated minimal alterations with exercise, although c-miR-133a (~4 fold, p = 0.049) and c-miR-149 (~2.4 fold; p = 0.006) were increased 4hr post-exercise. Thus a single bout of RE results in the increased abundance of a subset of miRs within the skeletal muscle, which was not evident in plasma. The lack a qualitative agreement in the response pattern of intramuscular and circulating miR expression suggests the analysis of circulatory miRs is not reflective of the miR responses within skeletal muscle after exercise.

## Introduction

Resistance exercise (RE) is the performance of muscle contractions with loads that are greater than would normally be encountered during activities of living [1]. RE stimulates transient increases in muscle protein synthesis, which when repeated over time in the form of resistance training, promotes muscle hypertrophy and enhanced contractile force as a result of increases in myofibre size and altered muscle architecture as well as adaptations in the extracellular matrix, tendons, innervating nerves and vascular tissue [2–4]. One element of this complex and coordinated adaptive response is post transcriptional regulation by microRNAs (miRs) [5]. Yet currently there remains very limited data on the role of miRs in muscle in response to RE. Few human studies have demonstrated acute alterations in miR-23a/b, -133b, -378 and 494 [6] as well as miR-1, -133a, -206, -208, -486 and -499 [7–10] following a single bout of RE. This set of miRs partially overlaps with those identified to respond to endurance exercise, where the predominant muscular adaption is increased mitochondrial oxidative capacity (although significant myofibre structural adaptations may also occur) [11, 12]. Acute endurance exercise and/or training has been shown to regulate a range of miRs including miR-1, -133a/b, -206, -23a/b and -378 [13–16]. Whilst these studies collectively demonstrate the impact of exercise on miR expression, it is likely that this represents only a fraction of what may be an intricately complex response.

Of the estimated 1881 miR species [17, 18] encoded in the human genome, studies to date have suggested a putative role for the involvement of approximately 30 miR species in the regulation of skeletal muscle function. Both *in-vitro* analysis of pathways related to muscle function and *in vivo* observations following changes in muscle contractile activity have identified miRs which may be involved in satellite cell proliferation; miR-1, -133a/b, -206, -486 [19–21], myogenic cell cycle regulation; miR-15a, -16 and -451a [22–24], myogenic differentiation; mir-1, -133a/b, -206, -486, -26a, -221 and -222 [7–9, 25–28] and fibre type determination; miR-208a/b and -499a [9, 29]. Similarly, further miRs have purported overlapping influence on skeletal muscle stress responsiveness, protein catabolism and atrophy, including miR-378a, -378b [30–32], miR-23a/b [33–38]-208a/b, -499a [7–9] and miR-23a/b [33–38]. Also implicated in the regulation of metabolism within myofibres, are miRs involved in the regulation of insulin and glucose responsiveness and signalling upstream of mTOR; miR-21 [39, 40], -148b [41] and -486 [42], Furthermore, miR-494 has been demonstrated to be involved in mitochondrial adaptation [43], whilst mir-126 [44, 45], -15a, 16 [46, 47] have been shown to be involved in angiogenesis within skeletal muscle. S1 Table shows the models used to identify each candidate miR.

miRs are known to act locally within the cells in which they are transcribed, but their existence in circulation indicates a potential to be released from one tissue type and act in another. *In vitro* analyses demonstrate miRs contained within skeletal muscle derived exosomes are detectible in the circulation [48, 49]. Additional evidence indicates that miRs contained in muscle derived exosomes can play a paracrine role in surrounding cell types [50]. As yet there is limited data on whether miR species expressed in skeletal muscle in response to exercise are also co-regulated in circulation following exercise. To date a limited number of studies have measured the muscle or plasma responses of a subset of miRs after RE but none have concurrently measured muscle and circulating miRs. Therefore, the aim of the current study was to characterize intra-muscular miR and c-miR responses during the early hours of recovery following an intense bout of high volume RE. For this, muscle biopsies and blood plasma were collected from the same participants at matching time-points. It was hypothesized that a relationship will exist between miR species that are regulated within skeletal muscle following exercise with the abundance of the same miR species in circulation.

## Methods

### Subjects

Nine healthy young men (18–35 years of age) were recruited for the study (Table 1). They were free of metabolic or neuromuscular diseases and any injuries which would impair their ability to perform RE. Inclusion criteria required that subjects were currently participating in a RE training program for ≥1 year which included at least one leg based training session per week. Participants were not taking any medication or performance enhancing drugs. Written consent was obtained before the commencement of study which was approved by the Northern Health and Disability Ethics Committee (New Zealand) (14/NTA/147).

**Table 1:** Subject characteristics.

Age (years)	24.6 ± 4.9
Height (cm)	181.0 ± 8.8
Weight (kg)	92.0 ± 10.6
BMI (kg/m^2^)	28.1 ± 3.3
Body fat (%)	18.0 ± 2.0
Isometric knee extension torque (Nm)	330.8 ± 28.9

Values presented as means ± SEM, n = 9.

### Experimental protocol

Approximately 4 weeks prior to the experimental trial day, participants performed a familiarization session which included one-repetition maximum (1RM) strength testing to determine the experimental exercise load (80% of 1RM). The maximal weight that subjects could lift for 3–6 repetitions (3–6RM) on a leg press and leg extension exercises was determined and participants’ 1RM was estimated using the Brzycki equation [51]. Participants were instructed to abstain from lower body RE for at least 48 hours prior to the experimental trial day. The evening before the trial, participants were asked to remain fasted from 10pm. The following morning, subjects arrived at (~7am) in a fasted state.

### Resistance exercise trial

Upon arrival at the laboratory, individuals rested in a supine position for ~30min prior to collection of resting muscle biopsy samples (see below). Participants then rested supine following collection of resting muscle biopsy for approximately ~5min after which the exercise protocol commenced. The exercise protocol began with two sets of ten repetitions of leg press with load increasing from (50–70% of 1RM) as warm-up. Participants then completed six sets of 8–10 repetitions of horizontal leg press, and eight sets of 8–10 repetitions of seated knee extensions at 80% of their predetermined 1RM. The exercises were performed with 2min rest between each set and exercise, with the final set of each exercise preformed to the point of momentary muscle fatigue. The exercise protocol took ~45min to complete. Following completion of the exercise protocol, participants rested in a supine position throughout the 4hr recovery period with additional biopsies collected at 2 and 4hr post exercise.

### Muscle biopsy sampling

Muscle biopsies (~100 mg) were collected from the *vastus lateralis* muscle under local anaesthesia (1% Xylocaine) using a Bergstrom needle modification of manual suction. All three biopsies were collected from the same limb starting proximally and moving distally. A gap of at least 2–3cm between sequential biopsies was maintained in order to avoid any potential confounding effects caused by repeated sampling for the same location. Biopsies were quickly frozen in liquid nitrogen and stored at -80°C until further analyses.

### Blood sampling

A cannula (20-gauge) was inserted into an antecubital vein and a baseline blood sample was obtained. A slow saline drip was used to keep the catheter patent. Further plasma samples were collected at two and four hours after the completion of the exercise bout. Plasma was collected in 10mL EDTA vacutainers and centrifuged immediately upon collection at 4°C at 1500g for 15min. The supernatant was collected in 1.6ml sterile tubes as 1ml aliquots and stored at -80°C.

### Muscle miR isolation

Total RNA was extracted from ~20mg of muscle tissue using the AllPrep^®^ DNA/RNA/miRNA Universal Kit (QIAGEN GmbH, Hilden, Germany) following the manufacturer's instructions. As per D’Souza et al. [52].

### Plasma miR extraction

Frozen plasma was thawed and centrifuged at 10,000g for 10min at 4°C. The top 200μL of plasma was transferred to a fresh tube. 10μl of 10mg/ml proteinase K was added (Macherey and Nagel, GmbH & Co. KG) and incubated at 37°C for 1hr. Samples were mixed in 3:1 Trizol LS (Thermo Fisher Scientific, Cat# 10296028, Carlsbad, CA, USA) vortexed and incubated at room temp for 5min. Next, 160μl of chloroform was added followed by 15sec of vigorous vortexing and a 10min room temp incubation. Samples were then centrifuged at 20,000g for 10min at 4°C. The upper aqueous phase was transferred to a new tube and mixed in equivolume (1:1) acid phenol chloroform (Thermo Fisher Scientific, Cat# AM9722, Carlsbad, CA, USA). After a 5min incubation, they were centrifuged again at 20,000g for 10min at 4°C. The upper aqueous phase was then transferred to a fresh tube and 4μl of glycogen was added as a carrier molecule and incubated for 5min. 1:1 volumes of 100% ethanol was then added to the samples. These were then applied to purelink columns (Thermo Fisher Scientific, Cat# 12183025, Carlsbad, CA, USA) and centrifuged at 3000g for 2min at room temp. Flow through was discarded and the process repeated if necessary. Samples were then washed twice in 500μl wash buffer II (Thermo Fisher Scientific, Cat# 12183025, Carlsbad, CA, USA) and centrifuged at 12,000g for 1min at room temp each time. The column filter was then dried by spinning at 20,000g for 3min at room temp with a fresh collection tube to ensure no contaminants were carried forward. Samples were then eluted in 30μl of 70°C RNAse free water, incubated for 5min and centrifuged at 12,000g at room temp. The eluate was reapplied to the column and centrifuged for a further 2min at 25°C. The exogenous spike-in cel-miR-39 was added prior to extraction (10pg) while cel-miR-238 was spiked-in prior to cDNA synthesis (10pg) to ensure variations in sample preparation were accounted for.

### Muscle and circulatory miR cDNA/RTPCR

10ng of total RNA from muscle and 2μl of input RNA from plasma (maximum suggested) was used for cDNA synthesis using TaqMan™ Advanced miRNA cDNA Synthesis Kit (Thermo Fisher Scientific, Carlsbad, CA, USA) as per manufacturers protocols. miR abundance were measured by RT‐PCR on a QuantStudio 6 (Thermo Fisher Scientific, Carlsbad, CA, USA) using Applied Biosystems Fast Advanced Master Mix (Thermo Fisher Scientific, Carlsbad, CA, USA).

Target miRs were as per Table 2 (Thermo Fisher Scientific, Cat# A25576, Carlsbad, CA, USA). The performance of probes were tested for linearity across an eight point standard curve with a 40 fold sample dilution range. Optimised sample dilutions were used to achieve product amplification (Ct) between 18–35 cycles. Ct values > 35 cycles were excluded from the analyses if duplicates had a standard deviation greater than 1 cycle. Final probe concentrations were maintained as suggested by the manufacturer in a 10μl reaction. Sample concentrations were 1:10 dilution of the stock pre-amplification generated product as per the manufacturers’ protocol. The geometric mean of three reference miRs (miR-361, -191 and -186) for muscle (p = 0.328) and five for plasma (miR-361, -191 and -186, -320a and -423) [53] (p = 0.690) were used for normalization based on miRs that showed the least variation amongst the current sample set. RTPCR data was analysed using 2^-ΔΔCT^ method. Where each subject was compared to themselves and pre exercise samples were given an arbitrary expression of 1 and post exercise values were represented as fold changes in respect to the pre exercise abundance [54].

**Table 2:** miRs analysed. Classification and identification number.

miR	ID number
**hsa-miR-23b-3p**	478602_mir
**hsa-miR-361-5p**	478056_mir
**hsa-miR-126-3p**	477887_mir
**hsa-miR-191-5p**	477952_mir
**hsa-miR-145-5p**	477916_mir
**hsa-miR-20a-5p**	478586_mir
**hsa-miR-149-5p**	477917_mir
**hsa-miR-146a-5p**	478399_mir
**hsa-miR-499a-3p**	477916_mir
**hsa-miR-26a-5p**	477995_mir
**hsa-miR-29b-3p**	478369_mir
**hsa-miR-486-5p**	478128_mir
**hsa-miR-451a**	477968_mir
**hsa-miR-208b-3p**	477806_mir
**hsa-miR-208a-3p**	477819_mir
**hsa-miR-206**	477968_mir
**hsa-miR-133b**	480871_mir
**hsa-miR-133a-3p**	478511_mir
**hsa-miR-1-3p**	477820_mir
**hsa-miR-222-3p**	477982_mir
**hsa-miR-221-3p**	477981_mir
**hsa-miR-494-3p**	478135_mir
**hsa-miR-454-3p**	478329_mir
**hsa-miR-378b**	479245_mir
**hsa-miR-378a-5p**	478076_mir
**hsa-miR-210-3p**	477981_mir
**hsa-miR-21-5p**	477975_mir
**hsa-miR-30b-5p**	478007_mir
**hsa-miR-148b-3p**	477806_mir
**hsa-miR-23a-3p**	478532_mir
**hsa-miR-16-5p**	477860_mir
**hsa-miR-15a-5p**	477858_mir
**hsa-miR-423-5p**	478090_mir
**hsa-miR-186-5p**	477940_mir
**hsa-miR-320a-5p**	478594_mir
**cel-miR-39-3p**	478293_mir
**cel-miR-238**	478292_mir

### Statistical analysis

A-priori sample size calculations were conducted using the average effect size and variance in all c-miRs regulated by resistance exercise as reported by Sawada et al. [55]. 8 participants were required to yield a statistical power of 80% and an additional participant was recruited to account for possible attrition. One way repeated measures ANOVA was carried out using SigmaPlot for Windows version 12.1 (Systat 218 Software Inc., San Jose, USA). Normality was determined using Shapiro-Wilk analysis. Holm-Sidak post hocs were used where appropriate to compare post exercise 2hr and 4hr time points against pre exercise values with alpha set at p≤0.05. Graphs were made using GraphPad Prism 7.00 Software (GraphPad Software Inc., La Jolla, CA). Data are shown as mean ± SEM.

## Results

### Muscle miRs

**Fig 1:**
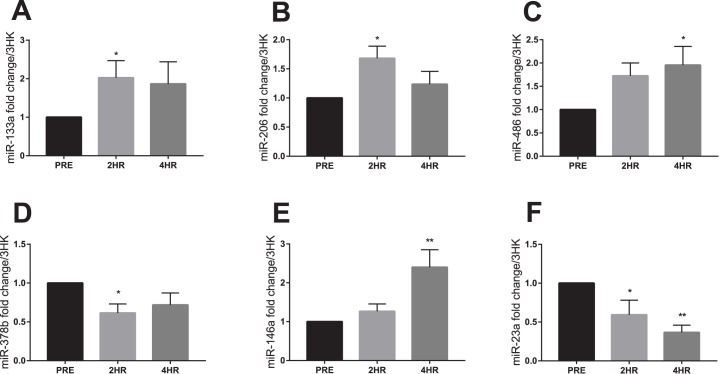
Skeletal muscle miR fold change to pre exercise. (**A**) miR-133a, (**B**) miR-206, (**C**) miR-486, **(D)** miR-378b, **(E)** miR-146a and **(F)** miR-23a expression normalized to geomean of 3 endogenous stable miRs. (Significant changes from baseline represented as * p≤0.05, ** p<0.01 and *** p<0.001, trends from baseline 0.05<p<0.1 represented as Φ). Data expressed are expressed as means ± SEM.

Of the 30 miRs analyzed in the biopsied skeletal muscle, miR-133a, -206, -486, -378b, 146a and -23a were regulated in response to the exercise bout. Of these, miR-133a and -206 were elevated at 2hr (p = 0.047 and p = 0.026 respectively) while miR-486 and -146a were increased at 4hr (p = 0.041 and p = 0.002 respectively) (Fig 1). miR-378b expression was reduced at 2hr (p = 0.010) while trending to remain lowered at 4hr (p = 0.051). Further, miR-23a was reduced at both 2hr and 4hr (p = 0.039 and p = 0.003 respectively). miR-1 approached significance with p = 0.059 as seen in Table 3.

**Table 3:** Muscle and plasma miRs expression fold change to baseline.

	Muscle			Plasma		
miR	2hr	4hr	p-value	2hr	4hr	p-value
mir-1	1.30±0.12	1.02±0.18	0.059	ND	ND	ND
mir-133a	1.25±0.44*	1.63±0.58	0.05	2.83±0.82	3.97±1.16*	0.049
mir-133b	1.50±0.34	1.39±0.53	0.221	2.55±0.67	1.97±1.02	0.286
miR-206	1.55±0.22*	1.10±0.23	0.027	1.56±0.45	2.10±0.88	0.418
mir-208a	1.98±0.45	1.46±0.45	0.13	1.02±0.26	1.06±0.45	0.872
mir-208b	1.46±0.20	1.37±0.22	0.139	ND	ND	ND
mir-486	1.73±0.28	1.78±0.39*	0.028	1.31±0.66	0.77±0.23	0.672
mir-499a	1.49±0.24	1.24±0.17	0.167	ND	ND	ND
mir-378a	1.33±0.15	1.19±0.20	0.178	0.96±0.23	1.11±0.50	0.926
mir-378b	0.61±0.12*	0.72±0.15 Φ	0.009	0.95±0.10	0.93±0.20	0.845
mir-23a	0.59±0.19*	0.37±0.09*	0.005	0.83±0.15	1.14±0.28	0.585
mir-23b	1.33±0.16	1.65±0.44	0.186	1.87±0.74	1.08±0.39	0.46
mir-15a	0.80±0.24	0.70±0.13	0.631	0.84±0.16	0.62±0.19	0.184
mir-16	0.76±0.17	1.04±0.20	0.365	0.85±0.19	0.62±0.12	0.08
mir-126	1.51±0.16	1.60±0.34	0.12	1.78±0.81	1.14±0.31	0.547
mir-148b	0.98±0.07	1.28±0.23	0.66	1.38±0.46	1.23±0.24	0.604
mir-30b	1.28±0.16	1.13±0.20	0.276	1.84±0.90	1.11±0.50	0.606
mir-21	1.08±0.11	1.09±0.17	0.616	2.24±0.82	0.93±0.20	0.207
mir-210	1.11±0.17	1.19±0.27	0.375	1.11±0.44	1.08±0.25	0.604
mir-221	1.40±0.34	1.69±0.30	0.219	2.04±0.78	1.79±0.48	0.471
mir-222	1.56±0.34	1.72±0.29	0.101	1.00±0.24	1.28±0.20	0.383
mir-454	1.94±0.41	3.28±1.67	0.169	1.71±0.76	1.24±0.50	0.49
mir-494	1.32±0.27	1.54±0.48	0.222	1.52±0.53	0.99±0.31	0.496
mir-451a	1.06±0.22	1.98±0.89	0.28	1.07±0.51	0.93±0.22	0.978
mir-29b	1.25±0.16	1.06±0.17	0.504	2.57±1.09	1.48±0.43	0.283
mir-26a	1.28±0.15	1.28±0.17	0.179	0.94±0.22	1.03±0.19	0.899
mir-20a	1.35±0.2	1.40±0.41	0.39	1.37±0.36	1.03±0.19	0.496
mir-145	1.16±0.18	1.27±0.25	0.379	1.38±0.67	0.77±0.21	0.609
mir-146a	1.17±0.19	2.14±0.47*	0.024	2.18±0.59	1.26±0.30	0.286
mir-149	1.58±0.28	2.02±0.58	0.15	1.67±0.44	2.38±0.28*	0.034

Values presented as means ± SEM, n = 9 and 7 for muscle and circulation respectively. P-values generated from One Way Repeated Measures ANOVA. ND indicated values for miRs that were not detected.

* indicates time points significantly different from respective baseline with p≤0.05 and Φ indicates trends from baseline 0.05<p<0.10.

### Circulatory miRs

**Fig 2:**
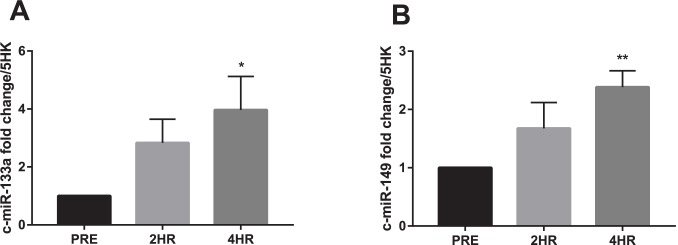
c-miR fold change to pre exercise. (**A**) c-miR-133a and (**B**) c-miR-149 expression normalized to geomean of 5 endogenous stable miRs (Significant changes from baseline represented as * p≤0.05, ** p<0.01 and *** p<0.001, trends from baseline 0.05<p<0.1 represented as Φ). Data expressed are expressed as means ± SEM.

Both miR-133a and -149 demonstrated significant elevations from baseline (p = 0.049 and p = 0.006) at 4hr following exercise (Fig 2). miR-16 was found to trend toward significance (p = 0.080) (Table 3). Of the other analysed miRs, miR-1 -208a and -499a were not detected in the present analyses.

## Discussion

Following a bout of RE, 6 of 30 miRs analysed exhibited altered expression within skeletal muscle, whilst just 2 of the 30 were altered in plasma within the 4 hour recovery period. Of these measured miR species, selected on the basis of prior evidence indicating *in-vivo* association, or *in-vitro* regulation of skeletal muscle mass or metabolic function, only miR-133a had increased abundances in both muscle and circulation. Thus, in the current experimental protocol, it is not evident that the analysis of circulating miRs is reflective of the altered miR expression within skeletal muscle after a single bout of RE. Hence, it is unlikely that the analysis of circulatory miRs in exercise recovery can be used as a ‘liquid’ biopsy or a proxy for alterations in muscle miR expression and therefore for signalling events being undertaken in skeletal muscle.

The current study demonstrated a number of unique findings, with respect to the skeletal muscle miR expression. Of the measured 30 miR species, it was shown that miR-133a and -206 were elevated at 2hr, with miR-486 and -146 being elevated at 4hr. It was also demonstrated that miR-378b was reduced at 2h, with miR-23a being reduced at both 2 and 4hr following RE.

The miR species elevated following a bout of RE (miR -133a, -206, -486 and -146a) have previously been analysed in skeletal muscle [7, 8, 37, 56]. Drummond et al. reported miR-133a and -206 were unaltered by RE [56]. While the cohort from the Drummond study were untrained and underwent co-ingestion of essential amino acids, the present study used a resistance-trained cohort, lack of feeding, and a larger total exercise volume, it is unclear which of these factors accounts for the discrepancy between studies. Drummond et al. [56] also report a decrease in miR-1 at 3 and 6h following exercise. Though not significant, the present study reports a trend for miR-1 to increase at 2hr before returning to baseline levels at 4hr. We show miR-486 to be unchanged at 2hr and elevated ~70% above baseline, 4hr following exercise. miR-486 abundance has previously been shown to increase in muscle 2hr following exercise amongst young men [10]. It is possible that either the training status of the participants or the much higher exercise volume used in the current study acted to delay or blunt the miR-486 elevation following exercise however, the mechanism of this is unclear. A blunting of the muscle protein synthetic response [57], satellite cell activation [57, 58] and IL-6 synthesis [59] has been reported following an acute bout of RE in trained individuals. We have recently shown baseline differences in muscle miR expression at rest between trained and untrained individuals [52]. Therefore, it is conceivable that training would also regulate the acute miR response to exercise. Increased miR-146a was reported within muscle at 4hr following exercise. miR-146a is reported as a negative regulator of muscle fibrosis in an animal model via inhibition of TGF-β signalling [60]. TGF-β signalling plays important roles in both hypertrophy [61] and atrophy [62] regulation suggesting a complex role for miR-146a following RE.

Several miR species were demonstrated to be acutely suppressed following the bout of RE. miR-378b which was reduced at 2hr, yet approached basal levels by 4hr post-exercise. miR-378 in cancer and cardiomyocyte models inhibit mitogen activated protein kinases (MAPK), p38 MAPK and ERK1/2 kinase signalling [63, 64]. These stress-related pathways have been reported to be activated by physical activity, with peak activity typically evident in the first few hours of exercise recovery [65–68]. In agreement with the time course of miR-378b expression, we report, phosphorylation of kinases within the MAPK and ERK pathway typically returns to pre-exercise levels within 4 hr of recovery [66, 69].

miR-23a was found to be significantly downregulated at both 2 and 4hr following exercise while miR-23b was unaltered. miR-23a and -23b inhibit MuRF1 and Atrogin1 dependent proteolytic signalling [35]. In contrast to our findings, Camera et al. [13], previously reported that muscular expression of both miR-23a and -23b were elevated after concurrent exercise in conjunction with protein feeding in untrained men. Taken together with the present study, these data suggest protein feeding following RE may regulate miR-23a/b expression which in turn may control catabolic signalling via MuRF1 and Atrogin1. This fits with what is known about muscle protein metabolism following RE. When performed in the fasted state, RE elevates MPB proportionally to MPS while total muscle protein balance remains negative [70]. Whereas when RE is combined with feeding, MPB is suppressed and accretion of muscle protein occurs.

Of the selected miRs, only 2 of the measured miRs were found to be altered in circulation this is in contrast to Margolis et al who found 9 c-miRs were altered 6hr following exercise in untrained men [71]. Of these, there was a ~4 fold increased in c-miR-133a at 4hr after exercise. Following exercise, both muscle and circulating miR-133a increase which agrees with its potential role as a muscle damage marker [72, 73]. Previously, circulatory expression of miR-133a has been observed to be increased after damaging exercise including resistance training [14, 73–76]. Further, myopathies including muscular dystrophy, where muscle damage is increased, also demonstrate increased c-miR-133a [75]. Similar increases in c-miR-133a are also evident with downhill running, but not for uphill running, where the extent of muscle damage differs markedly [77]. Previously, c-miR-133a was shown to be unchanged in untrained men following RE [55], while it was found to be downregulated immediately after exercise in trained men before returning to baseline levels 1hr after exercise [78]. This was inconsistent with our current results suggesting that the RE induced rise in c-miR-133a beginning at 2hr after exercise and may be reflective of muscle damage possibly due to the higher RE volume employed in the current study. Cui et al [78] report increased expression of miR-206 and -21 1hr following resistance exercise which is in contrast to our findings indicating no changes to these miRs following resistance exercise. The present study and the work conducted by Cui et al [78] differ in participant training status, exercise volume and the inclusion of upper body exercise. Multiple differences in research design make it difficult to account for discrepancies in results between studies.

In circulation, it was also demonstrated that c-miR-149 was increased 4hr post exercise. Previously, increased circulatory abundance has been reported 3 days, but not within the first 24hr, after RE [55]. Within skeletal muscle miR-149 promotes mitochondrial biogenesis by inhibiting PARP-2 [79]. c-miR-149 expression is reduced in coronary artery disease, suggesting a possible relationship to the vascular or cardiac responses to exercise [80]. Although the time course and functional role of c-miR-149 elevations following RE needs to be further elucidated it seems clear that acute RE regulates c-miR-149 regardless of participant training status.

While skeletal muscle is thought to secrete transcribed miRs into circulation via exosomes [14], the tissue of origin of c-miRs cannot be determined. Cell culture evidence shows c-miR abundance is downregulated with inhibition of exosomal secretion [81]. Although skeletal muscle is the primary tissue stimulated by RE, c-miR increases after RE could also be attributed to blood cells and other tissues capable of secreting exosomal miRs into circulation [82–84]. Circulatory miRs exist primarily bound to carrier proteins [85–87] rather than within exosomes. The current plasma extraction technique employed isolated total miRs. The inability to differentiate between exosomal and non-exosomal miRs could mask potentially important differences in exosomal or protein bound miR abundances. The role of miRs in circulation as long distance signalling molecules has been previously suggested [88, 89]. However, currently no relationship between muscle and plasma responses of individual miRs was demonstrated.

In conclusion, the findings of the current study demonstrate acute changes in both muscle and c-miR abundance following high volume RE in young strength trained men. By focusing on the early recovery phase, we identify miRs potentially involved in pathways associated with MPS rather than myogenesis and muscle repair. The utilization of a fasted model during recovery prevented confounding that may be regulated by nutrient dependent signalling. Our data suggest that both training status and nutritional state are important factors in determining the post exercise miR response. While some miRs appear to be globally responsive to exercise, exercise modality and intensity are important factors in the regulation of miRs. Additionally, our findings do not indicate a relationship between intramuscular and total c-miR abundances after RE. The measurement of miRs contained in circulating exosomes may better reflect muscle miR expression following exercise and should be further elucidated.

## Supporting information

S1 File Raw data file. Processed fold change data for each miR reported.
(XLSX)

S1 Table Evidence for selected miR subset. List of relevant references for each miR including experimental model and main findings.
(DOCX)
