# Supplementary material for: Acute resistance exercise modulates microRNA expression profiles: Combined tissue and circulatory targeted analyses
Source: PLoS One. 2017 Jul 27;12(7):e0181594. doi: 10.1371/journal.pone.0181594 (PMC5531502; doi:10.1371/journal.pone.0181594)
Supplement: S1 Table — List of relevant references for each miR including experimental model and main findings. (DOCX) [file pone.0181594.s002.docx]

**Table S1.** Model details for studies used to identify candidate miRs.

| Reference | Authors | Year | Organism | Model | Cell type | Manipulation | miR | Finding |
| --- | --- | --- | --- | --- | --- | --- | --- | --- |
| 7 | McCarthy and Esser | 2007 | Mouse | in vivo | skeletal muscle | synergist ablation | miR-1, -133a | miR-1, -133a decreased during skeletal muscle hypertrophy |
| 8 | Small et al | 2010 | Rat | in-vitro | neonatal rat cardiomyocytes (CMCs) | infected with viruses expressing MRTF-A or β-galactosidase | miR-486 | inverse correlation between miR-486 expression and PTEN in maturing heart |
| 9 | van Rooij et al | 2009 | Mouse | in-vivo | Skeletal and cardiac muscle | Wild type vs miR-208 deficient mice | miR-208a,-208b, -499 | Involvement of miR-208a,-208b and -499 in regulation of fiber type |
| 10 | Zacharewicz et al | 2014 | Human | in vivo | skeletal muscle | Acute resistance exercise (before and 2hr) | miR-486, -149 | miR-486 increased and -149 decreased in young men |
| 13 | Camera et al | 2016 | Human | in vivo | skeletal muscle | Acute concurrent exercise with placebo or protein feeding | miR-9, -23a/b, -133b, -181,-378 and -486 | increased miR-9, -23a/b, -133b, -181,-378 and -486 4h after exercise with protein but not placebo |
| 14 | Nielsen et al | 2014 | Human | in vivo | blood | Acute bouts with 12 week chronic training (aerobic) |  | 8 miRs acutely down regulated, 5 were upregulated 1h post with 1 miR upregulated 3h post exercise. Additionally chronic training reduced basal expression of 7miRs and increased expression of 2miRs |
| 15 | Nielsen et al | 2010 | Human | in vivo | skeletal muscle | Acute bouts with 12 week chronic training (aerobic) |  | miR-1 and -133a increased 60min post exercise prior to training period. No change in miR expression with acute exercise following 12 wk training period |
| 16 | Russell et al | 2013 | Human | in vivo | skeletal muscle | Acute aerobic exercise pre and post 10days of training | miR-1,-133a,-133b | miR-1,-133a,-133b increased acutely. 10days of training increased miR-1 and 133b compared to pretraining levels. |
| 19 | Chen et al | 2010 | Mouse | In-vitro | C2C12 myoblasts | Knockdown of miR-1 and -206 | mir-1, -206 | Knockdown of miR-1 and -206 increases satellite cell proliferation |
| 20 | Koutsoulidou et al | 2011 | Human | In-vitro | Feotal or new born infant myoblasts | Development | miR-1,-133a,-133b,-206 | miR-1, miR-133a, miR-133b and miR-206 are induced during human muscle cell differentiation and their levels are increased proportionally to the stage of muscle foetal development |
| 21 | Dey et al | 2011 | Mouse | In-vitro | C2C12 myoblasts | Different anti-miR treatments for miR proof of function | miR-206, -486 | miR-206 and -486 downregulate Pax7 by directly targeting its 3′ UTR |
| 22 | Bandi et al | 2011 | Human | In-vitro | NSCLC cell lines A549, H2009, H1299 and H358 | Transfection with miR-15a/16 and miR-34a | miR-15a/16 | miR-15a/16 and miR-34a act synergistically to induce arrest in G1-G0 |
| 23 | Musumeci et al | 2011 | Human | In-vitro | Prostate cancer cell lines isolated from surgical specimens | Transfection with miR-15a/16 | miR-15a/16 | miR-15 and miR-16 act as tumor suppressors both on tumor and on stromal cells |
| 24 | Nan et al | 2010 | Human | In-vitro | RCC-derived cell lines (786-O, A498, Caki-1 and Caki-2 and HK-2 | Over-expression of miR-451 | miR-451a | significantly impacts cell proliferation, invasion and apoptosis |
| 25 | Sander et al | 2008 | Mouse | In-vitro | murine MYC-induced lymphoma cell lines | MYC activation | miR-26a | demonstrate direct regulation of EZH2 via miR-26a |
| 25 | Sander et al | 2008 | Human | in vivo | PBMCs | Leukemia vs control | miR-26a |  |
| 26 | Lu et al | 2011 | Human | In-vitro | nasopharyngeal epithelial cell NP69, 5-8F, 6-10B, CNE1, CNE2, C666-1, HONE1, HNE, HEK 293T | upregulation vs knockout miR-26a | miR-26a | miR-26a functions as a growth-suppressive miRNA in NPC |
| 27 | Togliatto et al | 2013 | Mouse | In-vivo | skeletal muscle | Unilateral hindlimb ischemia | miR-221/222 | Treatement with pre-miR-221/222 results in increased number of PAX7/MyoD positive cells |
| 28 | Cardinali et al | 2009 | Quail | In-vitro | skeletal muscle | Myoblasts | miR-221/222 | miR-221 and miR-222 found to be modulated during myogenesis |
| 28 | Cardinali et al | 2009 | Mouse | In-vitro | Primary satellites and skeletal muscle | Primary satellite cells and two cell lines | miR-221/222 |  |
| 29 | McCarthy et al | 2009 | Mouse | In-vivo | skeletal muscle | Hindlimb suspension | miR-499, -208b | Reduction of miR-499 and -208b paralleled by upregulation of Sox6 and Purβ, known repressors of slow myosin expression, and a 28% decrease in β-MHC |
| 30 | McLean et al | 2015 | Human | In vivo | skeletal muscle | Acute aerobic exercise | mir-378 | 13 miRs differentially regulated with exercise |
| 31 | Davidsen et al | 2011 | Human | In vivo | skeletal muscle | 12 weeks chronic resistance exercise | mir-378,-451,-26a,-29a | expression of miR-378 and -451 was increased in low responders while -26a and -29a were decreased in low responders |
| 33 | Kapchinsky et al | 2015 | Human | In vivo | skeletal muscle | COPD patients vs age matched healthy controls | miR-145,-206 | miR-145 and -206 differentially expressed between groups |
| 34 | McCarthy | 2014 | Mouse | In vitro | skeletal muscle | transfection with miR-182 and dex treatment | miR-23a | Dex enhanced the exosomal packaging and release of two atrophy-related microRNAs (miR-1 and miR-23a) |
| 34 | McCarthy | 2014 | Rat | In vivo | skeletal muscle | STZ induced diabetes | miR-23a |  |
| 36 | Wang et al | 2016 | Human | In vivo | blood | AMI, CAD and healthy controls | miR-23a | elevated miR-23a in plasma was better than cTnI for reflecting the severity of coronary artery stenosis |
| 38 | Rezen et al | 2014 | Human | In vivo | skeletal muscle | 10 days bedrest | miR-23a, -23b | 15 mirR were downregulated with muscle following bedrest intervention including miR-23a and 23b |
| 39 | Zhong et al | 2013 | Mouse | In vivo | kidney tissue | Diabetes model | miR-21 | miR-21 in kidney cells enhanced, but knockdown of miR-21 suppressed, high-glucose-induced production of fibrotic and inflammatory markers. |
| 39 | Zhong et al | 2013 | Rat | In vitro | rat mesangial cell (MC) line, 1099, and tubular epithelial cell (TEC) line, NRK52E | Control vs TGF-β receptor II knockout model | miR-21 |  |
| 40 | Kornfeld et al | 2012 | Bats | in vivo | skeletal muscle | Hibernating atropy measures | miR-21 | expression of miR-21 decreased by 80% during torpor |
| 41 | Gastebois et al | 2016 | Human | in vitro | myotubes | inhibition of miR-148b expression, voluntary exercise suppression (inactivity) | miR-148b | overexpression of miR‐148b decreased NRAS and ROCK1 protein levels, and PKB phosphorylation and glucose uptake in response to insulin. |
| 41 | Gastebois et al | 2016 | Mouse | in vivo | skeletal muscle |  | miR-148b |  |
| 43 | Yamamoto et al | 2012 | Mouse | in vitro | C2C12 myoblasts | miR-494 knockdown | miR-494 | miR-494 knockdown upregulated the activity of mtTFA and Foxj3. |
| 44 | Wang et al | 2008 | Mouse | in vitro | endothelial cells | miR-126 null VS CONTROL | miR-126 | miR-126 is required for vascular integrity and angiogenesis, as well as survival post-MI, suggests that strategies to elevate miR-126 in the ischemic myocardium could enhance cardiac repair. |
| 44 | Wang et al | 2008 | Human | in vitro | umbilical vein endothelial cell (HUVEC) |  |  |  |
| 45 | Fish et al | 2008 | Human | in vitro | HeLa cells | Knockdown of miR-126 | miR-126 | miR-126 functioned in part by directly repressing negative regulators of the VEGF pathway, including the Sprouty-related protein SPRED1 and phosphoinositol-3 kinase regulatory subunit 2 (PIK3R2/p85-β). Increased expression of Spred1 or inhibition of VEGF signaling in zebrafish resulted in defects similar to miR-126 knockdown. |
| 45 | Fish et al | 2008 | Zebrafish | in vivo | developing zebrafish | reduced miR-126 expression during development | miR-126 |  |
| 46 | Yin et al | 2012 | Human | in vitro | umbilical vein endothelial cell (HUVEC) | cerebromicrovessel isolation, Hindlimb ischemia | miR-15a | miR-15a in ECs can significantly suppress cell-autonomous angiogenesis through direct inhibition of endogenous endothelial FGF2 and VEGF activities |
| 46 | Yin et al | 2012 | Mouse | in vivo | endothelial cells |  | miR-15a |  |
| 47 | Sun et al | 2012 | Human | in vivo | blood | transfected with miR-15a/16 | miR-15a/16 | Western blotting combined with the luciferase reporter assay demonstrated that VEGF-A was a direct target of miR-15a/16. Ectopic overexpression of miR-15a/16 led to decreased pro-angiogenic activity of MM cells. |
| 47 | Sun et al | 2012 | Human | in vitro | RPMI-8226, ARH-77, OPM-2, U266 and NIH929 (MM) cell lines |  | miR-15a/16 |  |
| 48 | Guescini et al | 2015 | Human | in vivo | Blood | Acute aerobic exercise trial | miR-133b,-181a | significant positive correlation was found between the aerobic fitness and muscle-specific miRNAs and EV miR-133b and -181a-5p were significantly up-regulated after acute exercise |
| 55 | Sawada et al | 2013 | Human | In vivo | blood | acute bout of resistance exercise | miR-146a,-149 | miR-146a and 149 were altered following exercise 1 -3 days later |
| 56 | Drummond et al | 2008 | Human | in vivo | skeletal muscle | Acute resistance exercise with protein feeding | miR-1, | acute protein anabolic stimulus, consisting of resistance exercise + EAA ingestion, has the ability to regulate miR-1 skeletal muscle expression in young men |
| 63 | Weng et al | 2016 | Human | In vitro | HCT-116, HCT-15, SW-480, SW-620, WiDr, HT-29 and Caco-2 colon cancer cell lines | transfected with miR-378 | miR-378 | BRAF mutants could even be inhibited in cell proliferation after elevated concentration of miR-378 in cells |
| 64 | Ganesan et al | 2013 | Rat | In vitro | Neonatal rat cardiomyocytes or fibroblasts | Cardiac hypertrophy model | miR-378 | miR-378 regulators cardiomyocyte hypertrophy, via suppression of MAPK signaling |
| 72 | Cacchiarelli et al | 2011 | Mouse | In vivo | Skeletal muscle and blood | Muscle and serum | miR-1,-133,-206 | propose that miR‐1, miR‐133, and miR‐206 are new and valuable biomarkers for the diagnosis of DMD and possibly also for monitoring the outcomes of therapeutic interventions in humans. |
| 72 | Cacchiarelli et al | 2011 | Human | In vivo | blood | Serum from dystropy patients | miR-1,-133,-206 |  |
| 73 | Gomes et al | 2014 | Human | In vivo | blood | Half marathon run - circulatory analysis | miR-1,-133,-206 | MiR-1, −133a, and −206 significantly increased after the race. These miRNAs are potential biomarkers of muscle damage or adaptation to exercise. |
| 74 | Cacchiarelli et al | 2010 | Mouse | In vitro | satellite cells | MDX model mice muscle was cultured | mir-1,-133a,-29c,-30c,-206,-23a | According to the mdx model, when dystrophin synthesis was restored, miR-1, miR-133a, miR-29c, miR-30c, and miR-206 increased, while miR-23a expression did not change |
| 75 | Mizuno et al | 2011 | Mouse | In vivo | MDX model | dystrophy model | miR-1,-133a,-206 | Found that the serum levels of several muscle-specific miRNAs (miR-1, miR-133a and miR-206) are increased in both mdx and CXMD |
| 75 | Mizuno et al | 2011 | Dog | In vivo | CXMDJ |  | miR-1,-133a,-206 |  |
| 77 | Banzet et al | 2013 | Human | In vivo | blood | Young men cross-over uphill running vs downhill running | miR-133b | hsa-mir-133b increased in response to eccentric exercise. |
| 79 | Mohamed et al | 2014 | Mouse | in vivo | skeletal muscle | Standard vs high fat diet | miR-149 | miR-149 is a regulator of SIRT-1/PGC-1α activation and provides a novel insight into skeletal muscle energy homeostasis |
| 80 | Ali Sheikh et al | 2015 | Human | in vivo | blood | CAD patients vs healthy control | miR-149 | circulating miR-149 levels were downregulated by 3.5-fold in stable CAD and 4.2-fold in unstable CAD patients, respectively. |
| 84 | Aoi et al | 2013 | Human | in vivo | blood | Acute and chronic human aerobic exercise trial | miR-486 | The reduction in circulating miR-486 may be associated with metabolic changes during exercise and adaptation induced by training |
